# Puerperal Eclampsia as a Result of Albuminuria in the Parturient State*Read by title before the Georgia Medical Association, April, 1896.

**Published:** 1896-10

**Authors:** A. A. Smith

**Affiliations:** Hawkinsville, Ga.


### PUERPERAL ECLAMPSIA AS A RESULT OF ALBUMINURIA IN THE PARTURIENT STATE.*

BY A. A. SMITH, M. D., HAWKINSVILLE, GA.

In the consideration of this subject it is not my purpose to advance any new theories as to its cause or treatment. The frequency with which we come in contact with puerperal convulsions, and the exceeding mortality as a result, requires the closest and most careful attention of every physician.

Under ordinary circumstances the presence of albumin in the urine is regarded as a symptom of serious organic disease, and experience shows that we have good reasons to look forward in anticipation of serious trouble in the future. There are other conditions, however, in which albumin is found in the urine during the pregnant and parturient state—in fact, according to the observations of a number of eminent authors, it is found to a greater or less extent in more than twenty per cent. of all pregnant women at some period during gestation. Again, we find it, temporarily, to a limited extent, as the result of certain articles of diet.

These latter conditions are not attended with any serious impairment of health, and, therefore, may not be regarded as symptomatic of any serious organic trouble.

The knowledge of the fact that these temporary conditions do exist in a number of cases without any lesions of the kidneys will cause us ofttimes to overlook the more serious forms of the trouble.

The existence of puerperal albuminuria is, as a rule, recognized only during the last months of pregnancy. It by no means follows, however, that this marks the period at which it first manifests itself in the urine. On the contrary, we may be certain that its recognition is often deferred to that period simply because the symptoms have not been such as to attract any particular attention. My experience and observation, therefore, lead me to the conclusion, that while in the greater number of instances we find the existence of the premonitory symptoms, viz., headache, dimness of vision, excessive œdema of the extremities, yet we find the occasional occurrence of a

*Read by title before the Georgia Medical Association, April, 1896.

case, when if these symptoms were present at all, they were so slight that they were passed by unnoticed.

In view of this fact, therefore, it is of the highest importance that each subject should be investigated at least once or twice a month for three months prior to the expected accouchement. Especially is this important if there should exist any premonitions of impending danger.

As to the cause which produces this abnormal change in the constituents of the urine, whether from the pressure of a gravid uterus, or from a reflex irritation of the kidneys, no attempt will be made to discuss now. The failure to eliminate the urea, owing to the disordered functions of the kidneys, and the blood thereby becoming poisoned with this effete matter, may be termed the predisposing causes. Other causes which have a direct effect in producing the paroxysm, and which may be designated as exciting causes, are the tension and excitability of the whole nervous system, together with the pain during labor.

The symptoms, as I have said, of the milder forms of the affection are either such as to attract no attention, or are simply looked upon as those which naturally arise during the pregnant state.

When, however, the dropsical effusion takes place, the swelling becoming general in the upper as well as lower limbs, our suspicions should become aroused and a careful investigation made, when the presence of the abnormal constituent (albumin) will be found in the urine. In the severer forms we find the extremities enormously swollen, a peculiar pallor of the face, wax-like color of the skin, and scanty secretion of urine; in fact, a condition of general anasarca.

These symptoms, together with others of lesser importance, mark the course of the more serious forms of albuminuria, and should, therefore, serve us as a note of warning of the almost inevitable danger. Thus we have this condition existing in varying intensity, the milder forms not of sufficient importance to attract the attention of the subject.

The health not being materially impaired, the symptoms, which are slight in character at first, are regarded as those naturally occurring in the ordinary course of pregnancy. Therefore, according to these observations, which have been made from an experience of twenty years, the following conclusions have been made, viz.: That in proportion to the quantity of albumin and the duration of its presence in the urine, just to the same extent will the poisonous effects upon the blood manifest itself in producing this condition with its attendant train of symptoms; and in the same ratio will the health and life of the subject be placed in jeopardy.

Seeing the great mortality resulting from this disease, both maternal and fœtal, and when we consider the fact that two lives are involved, thereby doubling our responsibility, it behooves us to watch closely and investigate each individual case coming under our care.

Puerperal albuminuria, not unlike all other maladies which we are called upon to treat, can be more effectually arrested or controlled when taken in its incipiency. In reference to the question of treatment, it is obvious that it must be of the greatest importance that the presence of albumin should be detected as early as possible after it has made its appearance in the urine. More especially is it of importance to be possessed of this information in order that we may adopt such measures as may remove, or at least mitigate, the symptoms before the period of labor arrives, at which time our experience and observation teach us to look forward with dread and apprehension to the convulsions and other alarming conditions which result from uræmic poisoning. In all cases when the symptoms, however slight, indicate even a remote possibility of albuminuria, it is necessary to make a test of the urine for albumin. The presence of this poisonous element, if slight and unaccompanied by any impaired condion of health, requires little or no treatment. In the event, however, that albumin is found in large quantities, and the consequent poisonous effects upon the blood in producing anæmia, dimness of vision, excessive œdema of the extremities, etc.,—these being the conditions indicating the more serious and malignant forms of this trouble—no time should be lost in these indications. Regarding the subject of treatment, our object should be to conduct the patient through to the successful termination of the pregnancy. For the accomplishment of this purpose, I know of nothing better than diuretics and tonics. Of the latter, the various preparations of iron seem to best subserve the purpose. Tonics, however, are only indicated when there are evidences of anæmia, such as have already been referred to. We sometimes meet with a subject, when the symptoms are urgent, such as violent headache, in which bloodletting is beneficial. This, however, affords only temporary relief in reducing the blood supply to the brain, and should be resorted to only in exceptional cases. When the case has advanced to that point where convulsions have already occurred, morphine, hypodermically, with chloral hydrate should be administered.

As already stated in the beginning of this paper, nothing new is advanced either as to the pathological conditions or the treatment. The main purpose is to urge the importance of carefully guarding our patients along this line, and I feel assured in so doing the mortality can be greatly reduced. The fact, as I have already said, of two lives in our hands should be a sufficient reason why we give these cases the closest of attention. Certainly we should do nothing less.

